# Is critical care ready for an economic surrogate endpoint?

**DOI:** 10.1186/s13054-015-0947-0

**Published:** 2015-06-11

**Authors:** M Elizabeth Wilcox, Gordon D Rubenfeld

**Affiliations:** Department of Medicine, University Health Network, Toronto Western Hospital, 399 Bathurst Street, Room 411M 2nd Floor McLaughlin Wing, Toronto, ON M5T 2S8 Canada; Interdepartmental Division of Critical Care, University of Toronto, 2075 Bayview Avenue, Room D503, Toronto, ON M4N 3M5 Canada; Department of Critical Care Medicine and Sunnybrook Research Institute, Sunnybrook Health Sciences Centre, 2075 Bayview Avenue, Room D108C, Toronto, ON M4N 3M5 Canada

## Abstract

Intensive care is expensive, and thus a body of research has focused on strategies to reduce its costs. However, efforts to reduce the total cost of intensive care have met with limited success, partly because of the challenges of calculating how much a day in the ICU actually costs. We discuss these challenges and introduce the concept of total cost savings as an outcome of critical care trials, assuming statistically negative effects on mortality and quality of life.

Healthcare is expensive, and intensive care is very expensive. ICUs are estimated to consume up to one-third of hospital costs and approximately 0.5 % of the US gross domestic product [1]. It is therefore not surprising that a lot of research has focused on strategies to reduce the cost of intensive care. Many studies have calculated the ICU cost by multiplying days in the ICU by cost per day, but what is truly surprising is the complexity of calculating the cost of a day in the ICU.

There are two common accounting formulas for healthcare costing, conveniently termed top down and bottom up. The top-down method takes the overall cost of care for an ICU divided by the number of patient-days of care delivered to calculate the average cost per day. Similarly, the bill that a hospital sends to a payer for an ICU day can be adjusted by some estimate of hospital overheads to obtain an idea of the average cost for that day. This method provides convenient numbers that are used frequently in the literature. For example, the cost-effectiveness study of antimicrobial-coated catheters used this approach to estimate that an ICU day cost US$1,152 in 1999, or roughly three times the cost of a hospital day [2]. In contrast, the bottom-up approach requires very granular cost data. Every bandage, catheter, lab test, and nursing and therapist hour must be accounted for. Even with this level of detail, assigning a dollar figure to these costs is challenging. A recent *Time* magazine article infuriated readers by reporting that hospitals charged US$18 for a glucose test strip that could be purchased at Amazon for US$0.55 [3]. Unlike the Amazon shopper, the hospital has to recoup the costs to order, store, track and, of course, bill for glucose strips. A tiny part of the hospital’s bill to dispose of hazardous wastes must also be built into the cost of glucose test strips. Of course, the hospital can decide to charge US$0.55 for glucose test strips, as long as it makes up these costs on aspirin or gauze. The rules for bottom-up accounting are quite complicated, and it is not clear how comparable these costs are between hospitals that might use different methods. Even when bottom-up data are available, the relative costs of an ICU day or a hospital day are determined by the room and board billing rate of the hospital [4]. Importantly, bottom-up and top-down approaches give different cost estimates [5].

Clinicians, researchers and policy-makers have found top-down numbers useful despite their limitations. For example, if catheter-related bloodstream infections extend the ICU length of stay by 4 days, then eliminating each infection should save the healthcare system US$4,608 (4 × US$1,152) in ICU costs. If a new sedative drug reduces the ICU length of stay by 0.5 days and the ICU treats 300 patients, the ICU should see an additional US$172,800 (0.5 × 300 × US$1,152) in the budget, minus the cost of the drug. However, while these average ICU costs and cost savings are easy to calculate and impressively large, they are deeply misleading. In this article we discuss some of the reasons why and explore the methods and rationale for studying total cost savings as a surrogate endpoint in critical care.

Reducing the total cost of hospital care is hard because most hospital costs are fixed, rather than variable. A detailed study of hospital costs at Cook County Hospital in the United States found that the majority of the costs were fixed and related to buildings, equipment, salaried labor and overheads [6]. In addition, variable costs tend to decrease as the length of stay increases; attempts to reduce costs by reducing variable costs such as length of stay or laboratory tests or medications have thus met with limited success. For example, significant reductions in hospital length of stay in the United States led to eventual increases in healthcare costs because patient care was simply shifted from hospitals to nursing homes and other post-acute care services [7]. A study evaluating reductions in length of stay in surgical patients similarly found small financial opportunities by eliminating the low-cost last days in the hospital [8]. The authors concluded that a focus on utilization earlier in the course of admission would yield greater cost savings.

There are three important reasons why reducing the length of stay in the ICU may not necessarily lead to overall cost savings. The first reason is that the variable costs of the days or hours that are saved at the end of the ICU stay are small compared with the first days [9]. In fact, the last days in the ICU have similar variable costs to the first days on the ward [10]. An analysis that assigns average costs to these inexpensive days therefore grossly overestimates the potential savings for reductions in length of stay. Second, depending on the reimbursement strategy and perspective of the cost analysis, reducing the ICU length of stay can paradoxically increase total ICU costs, because the new patient replacing the one leaving the ICU will require more intense and expensive care. Increasing access to ICU beds for patients who require them, and decreasing wait times in the emergency department, ward or post-anesthetic care unit is clearly a positive outcome, but it may not be a cost-saving one from the ICU perspective. Finally, if an intervention reduces the ICU length of stay by tacking on an equal or greater number of hospital days, the cost reduction will be significantly less than that calculated by a focus on ICU average costs.

In a recent issue of *Critical Care*, Turunen and colleagues explore the economic benefits of dexmedetomidine compared with standard sedatives in reducing days of mechanical ventilation [11]. The pharmaceutical company that markets the drug performed this economic analysis, using data from the Dexmedetomidine Versus Midazolam for Continuous Sedation in the Intensive Care Unit (MIDEX) and Dexmedetomidine Versus Propofol for Continuous Sedation in the Intensive Care Unit (PRODEX) trials, to measure cost savings attributable to dexmedetomidine. Readers who were skeptical of the two trials published in *Journal of the American Medical Association* because they thought that midazolam was an inappropriate comparator will be equally skeptical of this analysis. Careful readers will note that this study finds a statistically significant reduction in ICU length of stay whereas the parent trials did not, because it used different assumptions in counting the length of stay for patients who died [12].

However, there are some strengths to Turunen and colleagues’ study that can serve as a guide for future investigations. First, the authors account for the fact that different days in the ICU cost different amounts. They used a hybrid of activity-based bottom-up cost accounting to measure nursing activity using the Therapeutic Intervention Scoring System to estimate, for example, that a day on invasive mechanical ventilation cost 33 % more than a day not ventilated. Second, several sensitivity analyses were performed to see whether costing assumptions changed the results. Dexmedetomidine saved money in all of the scenarios; but in some, particularly in comparison with propofol, the savings are quite small and must be considered against the increased rates of bradycardia and hypotension.

The real question for intensivists to consider is posed by the article’s claim that if dexmedetomidine reduces the ICU length of stay by 30 h, then the same ICU staff could care for 19 extra patients for every 100 patients treated with the drug. If true, this important effect might let you treat an additional 80 patients per year in a typical 10-bed ICU. The problem with this conclusion is that you could do it with the same staff. In most ICUs, the last 30 h of an 8-day stay is provided by fewer nurses, but in this example 80 of those last days will be traded for new high-acuity first days of care. Unless the ICU uses a fixed staffing ratio for patients regardless of acuity, it is unlikely that the same staff could deliver this additional care, because it is different care.

Critical care has struggled with meaningful surrogate endpoints for trials [13]. Cost-minimization, the analysis presented here, assumes that the considered treatments have equivalent effects on patient-centered outcomes such as mortality and quality of life. This strategy overcomes a problem inherent in all nonmortality outcomes in critical care, namely informative censoring from mortality [14]. For example, both early death and successful weaning can reduce the duration of mechanical ventilation in a weaning trial, but we routinely ignore this and focus on duration of mechanical ventilation. Cost-minimization analysis addresses the problem of differences in outcomes by assuming they do not exist. This assumption has resulted in cost-minimization analysis being largely replaced by cost-effectiveness models that capture the uncertainty in outcomes from statistically negative clinical trials rather than ignoring them [15, 16].

Regardless of the technique, we believe that the research question posed by this study is fundamental to critical care. In the absence of compelling effects on mortality or long-term quality of life, the only valuable surrogate outcome in critical care might be safe and significant reductions in total cost. This outcome will capture much of what we already look for in our surrogate outcomes of duration of mechanical ventilation, organ failure, rescue therapies and length of stay, and will present them in a persuasive form to society and patients. Critical care is a long way from adopting this outcome. We need to develop understandable methods to express the uncertainty in safety and outcomes, reliable techniques to address informative censoring by death, and accurate accounting that includes post-ICU costs, international variations in labor and resource costs, and adequately addresses the issue of declining variable costs over time. Finally, and, most importantly, we need to see whether these modeled cost savings result in efficiencies that benefit our patients.
